# Group-level comparison of brain connectivity networks

**DOI:** 10.1186/s12874-022-01712-8

**Published:** 2022-10-17

**Authors:** Fatemeh Pourmotahari, Hassan Doosti, Nasrin Borumandnia, Seyyed Mohammad Tabatabaei, Hamid Alavi Majd

**Affiliations:** 1 Department of Biostatistics, Faculty of Paramedical Sciences, Shahid Beheshti University of Medical Sciences, Tehran, Iran; 2 Department of Mathematics and Statistics, Macquarie University, Macquarie, Australia; 3 Urology and Nephrology Research Centre, Shahid Beheshti University of Medical Sciences, Tehran, Iran; 4 Department of Medical Informatics, Faculty of Medicine, Mashhad University of Medical Sciences, Mashhad, Iran

**Keywords:** Connectivity, Subject heterogeneity, fMRI, Statistical power, Type I error rate

## Abstract

**Background:**

Functional connectivity (FC) studies are often performed to discern different patterns of brain connectivity networks between healthy and patient groups. Since many neuropsychiatric disorders are related to the change in these patterns, accurate modelling of FC data can provide useful information about disease pathologies. However, analysing functional connectivity data faces several challenges, including the correlations of the connectivity edges associated with network topological characteristics, the large number of parameters in the covariance matrix, and taking into account the heterogeneity across subjects.

**Methods:**

This study provides a new statistical approach to compare the FC networks between subgroups that consider the network topological structure of brain regions and subject heterogeneity.

**Results:**

The power based on the heterogeneity structure of identity scaled in a sample size of 25 exhibited values greater than 0.90 without influencing the degree of correlation, heterogeneity, and the number of regions. This index had values above 0.80 in the small sample size and high correlation. In most scenarios, the type I error was close to 0.05. Moreover, the application of this model on real data related to autism was also investigated, which indicated no significant difference in FC networks between healthy and patient individuals.

**Conclusions:**

The results from simulation data indicated that the proposed model has high power and near-nominal type I error rates in most scenarios.

**Supplementary Information:**

The online version contains supplementary material available at 10.1186/s12874-022-01712-8.

## Background

The non-invasive method of functional magnetic resonance imaging (fMRI) identifies the changes of functional connectivity (FC) patterns between healthy and patient individuals by measuring blood-oxygen-level-dependent (BOLD) [1]. Studies indicated that altered topological patterns of brain connectivity are related to many neurological disorders, including autism, Parkinson’s disease, and Alzheimer’s disease. Therefore, functional connectivity analysis is performed to investigate the connectivity patterns among the brain regions of patients to determine the biomarkers of neurodegenerative disorders [2–4].

Group comparison of brain connectivity patterns is usually performed using two graph theory-based techniques. In these methods, the network is represented by a graph so that the brain regions are defined as nodes, and the correlation between them is defined as the edge [5–7]. The first method is based on comparing the edges individually. In this approach, the correlation coefficient across time series of all-region pairs is calculated to estimate the edges, then the hypothesis of equal FC patterns between healthy and patient groups is assessed using a group-level statistic. Since there are a large number of pairwise edge comparisons, multiple testing procedures such as false discovery rate (FDR) are required to control false positive noise [8, 9]. In the second method, the comparison of brain connectivity is based on the network of edges. Some models investigate the changes in the FC patterns as a subnetwork of the edges, and others investigate them as an entire network of the edges. However, fitting a proper model to determine differentially expressed FC characteristics between subgroups faces some problems due to the high dimension of connectivity data and their complex correlation structure [10–12].

Generally, the fit of the FC model based on the edge requires estimating the covariance matrix among the edges. Since the structure of dependency between the edges is related to the network topological structure of brain regions, considering this feature can accurately estimate the model parameters. In conventional statistical models, the structure of parameters dependency is often determined by the distance between brain regions. For example, spatial dependencies are defined based on the distance of voxels that belong to the same region of interest (ROI) [13–16].

However, because the dependency among the edges is defined based on the network topological structure and is not necessarily limited to spatial adjacency, the results of these models may not be appropriate. On the other hand, because of the high-dimensional FC data, it is difficult to estimate a large number of parameters in the sample covariance matrix between edges, especially if the number of regions in the brain is large. In this regard, there are several optimization techniques for estimating parameters when the number of variables is more than the sample size [17–21]; however, these methods may overlook the network topological features to control false positive and false negative noises [22, 23].

Another feature of FC data is the heterogeneity, which indicates the existence of differences in the connectivity structure of brain regions across subjects within the same group. By considering the heterogeneity in the model, the power of tests can be increased to diagnose neurodegenerative disorders associated with brain connectivity correctly [24]. The heterogeneity across subjects can indicate the changes in the performance of cognitive and behavioural domains in healthy individuals, as well as the severity of symptoms and different responses to clinical interventions in patients [25–28]. In this regard, using the penalized model-based clustering, DiLernia et al. (2020) reported distinct FC patterns for each individual in the healthy and patient [29].

Many studies have been conducted on FC modelling, which has considered some of the FC data properties. For example, Fiecas et al. (2017) accounted the heterogeneity across subjects by introducing a variance component model. However, in addition to ignoring the spatial features of the brain network, their proposed model cannot estimate the parameters of the edges when the number of ROIs is large. Furthermore, given the large number of ROIs in many brain atlases, applying this model for more than 20 ROIs faces computational challenges [24]. On the other hand, Chen et al. (2020) proposed a nonparametric Bayesian model to estimate the massive parameters of the covariance matrix among edges, which takes the spatial network feature in terms of its topological structure. However, the effect of between-subject heterogeneity is not considered in this model, and equalization of the whole FC network is not examined [23]. Given these challenges, the present study proposes a more comprehensive model for examining differentially expressed FC characteristics between subgroups by considering the network topological structure and the heterogeneity across subjects.

## Materials and methods

### Estimating covariance matrix between edges

Connectivity data for each subject (*n* = 1, …, *N*) is formulated by a matrix ${\boldsymbol{M}}_{\mathrm{v}\times \mathrm{v}}^n=\left\{{M}_{i,j}^n\right\}$ so that the elements of ${M}_{i,j}^n$ represents the Fisher’s Z-transformed correlations between brain regions *i* and *j*. The entire brain connectivity network can be displayed by a graph ***M***^*n*^ = {*V*, *E*}, where *V* is the set of nodes and *E* = *V*(1 − *V*)/2 is the set of edges. In this setting, the degree is determined by the number of edges, and the correlation coefficient is determined by the edges. The corrections have been made. The matrix ***M***^*n*^ is transformed into a vector ${\boldsymbol{Y}}_{\mathbf{1}\times \boldsymbol{E}}^{\boldsymbol{n}}$. Suppose that the vector **Y** follows a normal multivariate distribution ${\boldsymbol{Y}}_{\mathbf{1}\times \boldsymbol{E}}^{\boldsymbol{n}}\sim MVN\left({\boldsymbol{X}}_{\boldsymbol{n}}^{\boldsymbol{T}}{\boldsymbol{\beta}}_{\boldsymbol{p}\times \boldsymbol{E}},{\mathbf{\sum}}_{\boldsymbol{E}\times \boldsymbol{E}}\right)$, which ${\boldsymbol{X}}_{\boldsymbol{n}}^{\boldsymbol{T}}$ indicates the design matrix with p covariates, ***β***_***p*** **×** ***E***_ is the effect of covariates on the vector **Y**, and **∑**_***E*** **×** ***E***_ is the covariance matrix. The model has the following form:

1 $${\boldsymbol{Y}}_{\boldsymbol{N}\times \boldsymbol{E}}={\boldsymbol{X}}_{\boldsymbol{N}\times \boldsymbol{p}}^{\boldsymbol{T}}{\hat{\boldsymbol{\beta}}}_{\boldsymbol{p}\times \boldsymbol{E}}+{{\boldsymbol{R}}^{\boldsymbol{o}}}_{\boldsymbol{N}\times \boldsymbol{E}}$$

where ***R***^***o***^_***N*** **×** ***E***_ is the residual matrix. In the nonparametric Bayesian model, ***R***^***o***^_***N*** **×** ***E***_ is used as input data to estimate the covariance matrix by considering the characteristics of the network topological structure.

Let ***R***^***n***^_**1 ×** ***E***_ ∣ ***Λ***_***E*** **×** ***E***_~*MVN*(**0**, ***Λ***_***E*** **×** ***E***_). The edges correlation matrix ***Λ***_***E*** **×** ***E***_ = *f*(*G*, ***ρ***) is a function of the unknown network structure *G* and the correlation factors ***ρ*** = (*ρ*_0_, *ρ*_1_, …, *ρ*_*k*_). *G* is a probability measurement of the latent K networks that follows a Dirichlet process, with parameters *G*_*o*_ and *α*:

2 $$G\sim DP\left(\alpha, {G}_o\right)$$

The correlation matrix based on the network topological structure is given by the equation:

3 $$\Lambda_{e_{i,j}\times e_{i',j'}}=\left\{\begin{array}{llc} \rho_k && if \;\;\; \omega_i=\omega_j=\omega_i'=\omega_j'=C_k \cr \rho_0 && \ otherwise \end{array}\right.$$

${\varLambda}_{e_{i,j}\times {e}_{i^{\prime},{j}^{\prime}}}$ is indeed an element of the matrix ***Λ***_***E*** **×** ***E***_ which is determined by the correlation between the edges *e*_*i*, *j*_ and ${e}_{i^{\prime },{j}^{\prime }}$. Let *e*_*i*, *j*_ denote connectivity between regions *i* and *j*, *i* ≠ *j*. Based on whether region *i* belongs to the cluster *k* or not, the term *ω*_*i*_ = *C*_*k*_ is used as an indicator variable. If two edges are in the same cluster, it can be supposed that*:*

$${e}_{i,j}\cong {e}_{i^{\prime },{j}^{\prime }}\in {C}_k\left({e}_{i,j}\in {C}_k,{e}_{i^{\prime },{j}^{\prime }}\in {C}_k\right)\,\,\,\ if\ and\ only\ if\,\,\,\ {\omega}_i={\omega}_j={\omega}_{i^{\prime }}={\omega}_{j^{\prime }}={C}_k$$

The nonparametric Bayesian model is used to detect neighbourhood networks based on the input data and correlation structure in Eq. (3).

A discrete distribution is employed to assign each region in the K networks with probability ***π*** = (*π*_1_, …, *π*_*k*_).

The Dirichlet process in Eq. (2) is therefore equivalent to the following equations:

$${\omega}_i={C}_k \mid \boldsymbol{\pi} \sim Discrete\left(\boldsymbol{\pi} \right),i=1,\dots, V$$

$$\boldsymbol{\pi} \mid \alpha \sim Dirichlet\left({}^{\alpha }\!\!\left/ \!{}_{K}\right.,\dots, {}^{\alpha }\!\!\left/ \!{}_{K}\right.\right),k\to \infty$$

The distributions of *ρ*_*o*_ and *ρ*_*k*_ are assumed to be normal with parameters *μ*_*o*_ *، μ*_*k*_ *،* ${\tau}_o^2$ and ${\tau}_k^2$:

$${\rho}_o \mid {\mu}_o,{\tau}_o^2\sim N\left(\ {\mu}_o,{\tau}_o^2\right)$$

$${\rho}_k \mid {\mu}_k,{\tau}_k^2\sim N\left({\mu}_k,{\tau}_k^2\right),k=1,\dots, K$$

The posterior probability of ***R***_***N × E***_ is calculated by the prior distribution ***ρ***, ***ω*** and function *f*:

$$p\left({\boldsymbol{R}}_{\boldsymbol{N}\times \boldsymbol{E}}|{\boldsymbol{\varLambda}}_{\boldsymbol{E}\times \boldsymbol{E}},G,\boldsymbol{\rho} \right)p(G)p\left(\boldsymbol{\rho} \right)\propto {\left|2\pi {\boldsymbol{\varLambda}}_{\boldsymbol{E}\times \boldsymbol{E}}\right|}^{-\frac{N}{2}}\exp \left(-\frac{1}{2}\sum_s{\boldsymbol{R}}_{\boldsymbol{n}}^{\boldsymbol{T}}{\left({\boldsymbol{\varLambda}}_{\boldsymbol{E}\times \boldsymbol{E}}\right)}^{-1}{\boldsymbol{R}}_{\boldsymbol{n}}\right)p(G)p\left(\boldsymbol{\rho} \right)=\exp \left\{-{}^{N}\!\!\left/ \!{}_{2}\right.\left(\mathit{\log}{\left|{\boldsymbol{\varLambda}}_{\boldsymbol{E}\times \boldsymbol{E}}\right|}^{-1}+ tr\left(\boldsymbol{H}{\left({\boldsymbol{\varLambda}}_{\boldsymbol{E}\times \boldsymbol{E}}\right)}^{-1}\right)\right)\right\}p(G)p\left(\boldsymbol{\rho} \right)$$

Where ***H***^***o***^ = (***R***^***o***^)^*T*^***R***^***o***^/(*N* − *p*) and ***H*** = (*Diag*(***H***^***o***^))^−1/2^***H***^***o***^(*Diag*(***H***^***o***^))^−1/2^.

Then the sampling of the conditional posterior probabilities ***ω*** and ***ρ*** is obtained by the Markov chain Monte Carlo (MCMC) algorithm:

$$p\left({\omega}_i={C}_k|{\boldsymbol{\omega}}_{-\boldsymbol{i}},\boldsymbol{\rho}, {\boldsymbol{R}}_{\boldsymbol{N}\times \boldsymbol{E}}\right)$$

$$\propto \exp \left\{-N/2\right(\log \left(\mathit{\det}\left(f\left(\left({\boldsymbol{\omega}}_{-\mathbf{1}},{\omega}_i={C}_k\right),\boldsymbol{\rho} \Big)\right)\right)+ tr\left(\boldsymbol{H}f{\left(\left({\boldsymbol{\omega}}_{-\mathbf{1}},{\omega}_i={C}_k\right),\boldsymbol{\rho} \right)}^{-\mathbf{1}}\right)\right)\Big\}\times \frac{m_{- ik}}{\mathrm{v}-1+\alpha }$$

The number of nodes within the network k is showed by *m*_−*ik*_ = ∑_*j* ≠ *i*_*I*(*ω*_*j*_ = *C*_*k*_)*.*

$$p\left({\omega}_i\ne {\omega}_j\ for\ all\ j\ne i|{\boldsymbol{\omega}}_{-\boldsymbol{i}},\boldsymbol{\rho}, {\boldsymbol{R}}_{\boldsymbol{N}\times \boldsymbol{E}}\right)$$

$$\propto \exp \left\{-N/2\right(\mathit{\log}\left(\mathit{\det}\left(f\left(\left({\boldsymbol{\omega}}_{-\mathbf{1}},{\omega}_i={C}_{k+1}\right),\boldsymbol{\rho} \right)\right)\right)$$

$$+ tr\left(\boldsymbol{H}f{\left(\left({\boldsymbol{\omega}}_{-\mathbf{1}},{\omega}_i={C}_{k+1}\right),\boldsymbol{\rho} \right)}^{-\mathbf{1}}\right)\left)\right\}\times \frac{\alpha }{\mathrm{v}-1+\alpha }$$

The Sherman–Morrison formula is used in the computation of (***Λ***_***E × E***_)^***−*1**^ [30]*:*

$$f{\left(\boldsymbol{\omega}, \boldsymbol{\rho} \right)}^{-1}={\left({\boldsymbol{\varLambda}}_{\boldsymbol{E}\times \boldsymbol{E}}\right)}^{-\mathbf{1}}={\left(A+\sqrt{\rho_o}{\mathbf{1}}_{E\times 1}{\left(\sqrt{\rho_o}{\mathbf{1}}_{E\times 1}\right)}^T\right)}^{-\mathbf{1}}$$

$$={A}^{-1}-\frac{\rho_o{A}^{-1}{\mathbf{1}}_{E\times E}{A}^{-1}}{1+{\rho}_o{\mathbf{1}}_{E\times 1}^T{A}^{-1}{\mathbf{1}}_{E\times 1}}$$

$$p\left({\rho}_o|\boldsymbol{\omega}, \boldsymbol{H},{\boldsymbol{\rho}}_{-\mathbf{0}}\right)$$

$$\propto \exp \left\{-N/2\left(\log \left(\det \left(f\left(\boldsymbol{\omega}, {\rho}_o,{\boldsymbol{\rho}}_{-\mathbf{0}}\right)\right)\right)+ tr\left(\boldsymbol{H}f{\left(\boldsymbol{\omega}, {\rho}_o,{\boldsymbol{\rho}}_{-\mathbf{0}}\right)}^{-1}\right)\right)\right\}\times p\left({\rho}_o|{\mu}_o,{\tau}_o^2\right)$$

$$p\left({\rho}_k|\boldsymbol{\omega}, \boldsymbol{H},{\boldsymbol{\rho}}_{-\boldsymbol{k}}\right)$$

$$\propto \exp \left\{-N/2\left(\log \left(\det \left(f\left(\boldsymbol{\omega}, {\rho}_k,{\boldsymbol{\rho}}_{-\boldsymbol{k}}\right)\right)\right)+ tr\left(\boldsymbol{H}f{\left(\boldsymbol{\omega}, {\rho}_o,{\boldsymbol{\rho}}_{-\boldsymbol{k}}\right)}^{-1}\right)\right)\right\}\times p\left({\rho}_k|{\mu}_k,{\tau}_k^2\right)$$

Finally, given the posterior distributions of ***ω*** and ***ρ***, the covariance matrix between the edges is estimated. A detailed description of the theoretical methods of the Bayesian nonparametric model is available in the original paper [23].

### Estimating between-subject variability

This section describes the estimation of the covariance matrix **Ψ** to control subject heterogeneity and the estimation of edges parameters **β** by considering the dependence structure between edges **∑**_***E*** **×** ***E***_. The terms ***ψ***_**1**_***,*** …***,ψ***_***N***_ are considered to apply between-subject variability. It is assumed that ***ψ***_***N***_(1, …, *N*) is the scaled diagonal identity matrix with *E* × *E* dimensions. The **Ψ** covariance is a block-diagonal matrix with ***ψ***_**1**_***,*** …***,ψ***_***N***_ matrices along its diagonal. Each entry of ***ψ***_***N***_ indicates the variability that can be assigned to subject sampling.

Estimation of both **β** and **Ψ** parameters are obtained using the iterative method. In this process, an initial value of $\hat{\boldsymbol{\Psi}}$ is replaced in Eq. (4), and then the residuals **R** = **Y** − **Xβ** are calculated.

4 $$\hat{\boldsymbol{\beta}}={\left({\boldsymbol{X}}^{\prime }{\left({\hat{\boldsymbol{\Sigma}}}_{\boldsymbol{E}\times \boldsymbol{E}}+{\hat{\boldsymbol{\Psi}}}_{\boldsymbol{E}\times \boldsymbol{E}}\right)}^{-1}\boldsymbol{X}\right)}^{-1}{\boldsymbol{X}}^{\prime }{\left({\hat{\boldsymbol{\Sigma}}}_{\boldsymbol{E}\times \boldsymbol{E}}+{\hat{\boldsymbol{\Psi}}}_{\boldsymbol{E}\times \boldsymbol{E}}\right)}^{-1}\boldsymbol{Y}$$

The estimate $\hat{\boldsymbol{\varPsi}}$ to be updated using **R**. The process is repeated until convergence is achieved. To estimate the parameter **Ψ**, the outer product of the *E* × 1 residual vector is obtained for each subject, then averaged over these vectors to create a residual covariance matrix ${\hat{\boldsymbol{\Omega}}}_{\boldsymbol{E}\times \boldsymbol{E}}$. For ***ψ***_***N***_, the structures of a scaled identity matrix and compound symmetry with the forms ***ψ***_***N***_ = *σ*^2^*I* and ***ψ***_***N***_ = *σ*^2^ + *b* are assumed. Where *σ*^2^ is set as the average of all diagonal elements of ${\hat{\boldsymbol{\Omega}}}_{\boldsymbol{E}\times \boldsymbol{E}}-{\hat{\boldsymbol{\Sigma}}}_{\boldsymbol{E}\times \boldsymbol{E}}$, *I* is the identity matrix with dimensions of *E* × *E* and b is considered as the average of the off-diagonal elements of ${\hat{\boldsymbol{\Omega}}}_{\boldsymbol{E}\times \boldsymbol{E}}-{\hat{\boldsymbol{\Sigma}}}_{\boldsymbol{E}\times \boldsymbol{E}}$.

### Group-level statistic to detect differentially expressed FC patterns.

The basic hypotheses in this study are examined using the following test statistics.

**Hypothesis 1**: The entire functional connectivity networks are significantly different between case and control groups. The test statistic is:

5 $${\left(\boldsymbol{C}\left(\hat{{\boldsymbol{\beta}}_{\mathbf{1}}}-\hat{{\boldsymbol{\beta}}_{\mathbf{2}}}\right)\right)}^{\prime }{\left(\boldsymbol{C}\left(\hat{\operatorname{var}}\left(\hat{{\boldsymbol{\beta}}_{\mathbf{1}}}\ \right)+\hat{\operatorname{var}}\left(\hat{{\boldsymbol{\beta}}_{\mathbf{2}}}\right)\right){\boldsymbol{C}}^{\prime}\right)}^{-1}\left(\boldsymbol{C}\left(\hat{{\boldsymbol{\beta}}_{\mathbf{1}}}-\hat{{\boldsymbol{\beta}}_{\mathbf{2}}}\right)\right)$$

Where the contrast matrix of C is the *E* × *E* identity matrix. The parameters *β*_1_ and *β*_2_ are estimated according to Eq. (4). The covariance matrix for each group is estimated $\hat{\operatorname{var}}\left(\hat{\boldsymbol{\beta}}\right)={\left({\boldsymbol{X}}^{\prime }{\left({\hat{\boldsymbol{\Sigma}}}_{\boldsymbol{E}\times \boldsymbol{E}}+{\hat{\boldsymbol{\Psi}}}_{\boldsymbol{E}\times \boldsymbol{E}}\right)}^{-1}\boldsymbol{X}\right)}^{-1}.$

**Hypothesis 2**: The functional connectivity of the ROI pairwise is significantly different between the case and the control groups. The following test statistic is:

6 $${\left(\left(\hat{\beta_1}(e)-\hat{\beta_2}(e)\right)\right)}^{\prime }{\left(\left(\hat{\operatorname{var}}\left(\hat{\beta_1}(e)\ \right)+\hat{\operatorname{var}}\left(\hat{\beta_2}(e)\right)\right)\right)}^{-1}\left(\left(\hat{\beta_1}(e)-\hat{\beta_2}(e)\right)\right),e=1,..,E$$

**Fig. 1:**
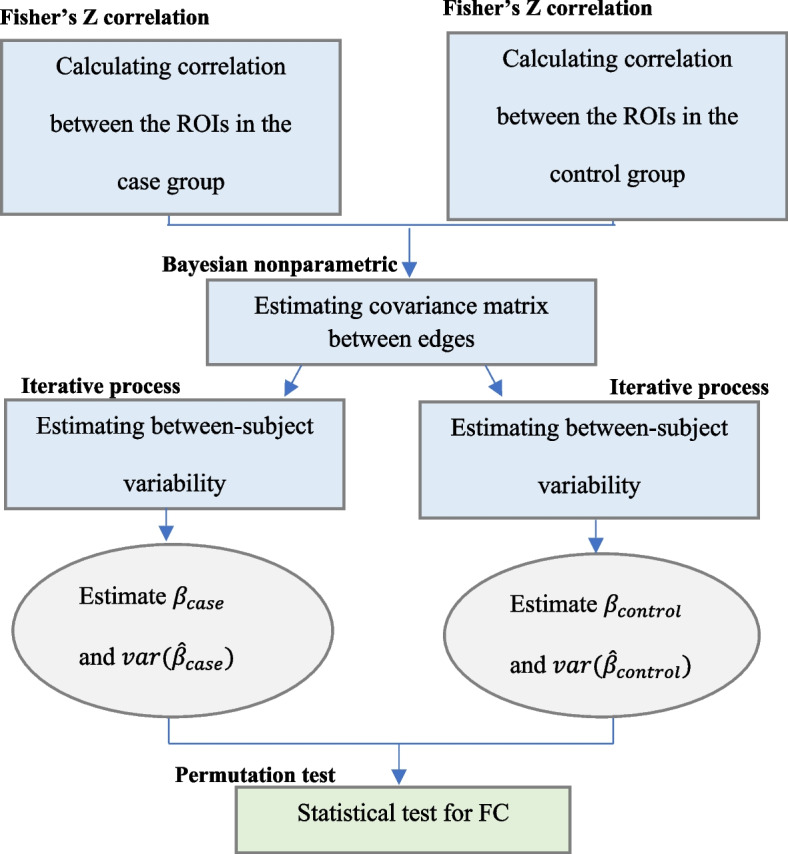
Process of the proposed approach to determine differentially expressed FC patterns between case and control groups

A permutation test has been used to check the hypotheses while controlling type I error. In this regard, with each resampling of subjects between two groups, the interest parameters are estimated using the iterative algorithm of section. The values of the statistics for each permutation are then derived by replacing them with Eq. (5) and (6). In addition, the FDR method was used to adjust the *p*-value of the permutation test since there was the high number of multiple comparisons in the second hypothesis. In both the simulation and real data assessments, the nominal level of statistical significance was set at α = 0.05. Figure 1 shows a process of the proposed method to determine differentially expressed FC patterns between two groups.

### Simulation settings

In this section, simulation data were applied to assess the performance of the proposed method in terms of statistical power and type I error. The number of nodes *V*= 20, 25, and 30 with two latent clusters were considered to simulate the dependence structure between the edges. The correlation of the edges inside the cluster was *ρ*_1_ = *ρ*_2_ equal to the values 0.3, 0.5, and 0.7 as well as the correlation of the edges outside the cluster was *ρ*_0_ = 0. In the next step, the *Uniform*(−*δ*, *δ*) distribution was employed to create subject-specific effects. The values for each subject were separately generated from this distribution and were added to the diagonal entries of the edges covariance matrix. Given that the *δ* parameter controls the degree of heterogeneity between subjects, *δ*= 0.15 and 0.3 were considered as low and high heterogeneity, respectively.

In the following, connectivity data of each group is generated using a multivariate normal distribution according to the features of the covariance matrix. For example, Fisher’s Z-transformed correlation is considered a connectivity metric.

$$Y_{N\times E}\thicksim \left\{\begin{array}{llc} MVN\left(0,{\sum}_{E\times E}+\kern0.5em \Psi \kern0.5em \right)+ d \;\;\;\;\; \mbox{Control group} \\\cr MVN\left(0,{\sum}_{E\times E}+\kern0.5em \Psi \right) \;\;\;\;\;\;\;\;\;\;\;\;\; \mbox{Case group}\end{array}\right.$$

The d vector reflects the differentially expressed FC network between groups so that an *E* × 1 zero vector is considered, and then *d* = 0.8 is randomly replaced for about 5% of zero values.

The results of simulation data were based on 100 iterations for each setting to estimate power and type I errors under the null hypothesis. For all tests, the permutation number was 500. The model performance was evaluated based on the amount of correlation between edges, heterogeneity, and the dimensions of communication data in the sample size of 10 and 25. The proposed methods were implemented with R software version 4.0.5 and MATLAB R2021b. The codes of the method are available upon request.

### Application to autism data

#### Subjects

This study examined resting-state fMRI data of 25 men with autism)7.29–15.66 years old) and 25 healthy men (7.26–14.66 years old) with a full intelligence quotient (FIQ) score above 87. The data were matched on age (*p* = 0.272) and FIQ score (*p* = 0.659).

#### Data acquisition

The resting-state fMRI data of subjects were obtained from the ABIDE database gathered by NYU Langone Medical Center [31]. fMRI images were collected using a 3 T Siemens Allegra scanner for 6 minutes. Participants were asked to relax and stare at the white cross displayed in the middle of a screen with a black background. The following is the scan acquiring procedure: TR = 2000 ms, TE = 15 ms, 33 slices with thickness = 4.0 mm, FOV = 240 mm, flip angle = 90.

#### Data processing

The pre-processed data were selected from the ABIDE dataset at http://fcon_1000.projects.nitrc.org/indi/abide/ [32]. Pre-processing of fMRI scans was performed using SPM8 software. The first ten values of each time series were eliminated due to the correction of the initial scan heterogeneity and the adaptability of individuals to the surrounding states; hence, a total number of 170 values per individual was considered. Normalization of scans was performed to the MNI (Montreal Neurological Institute) space with a resolution of 3 × 3 × 3 mm^3^ and correction of head movement according to Friston 24-parameter model [33] .According to the AAl (automated anatomical labelling) atlas, the pre-processed data were partitioned into 116 regions. [34]. The number of regions *V* = 30 affected by autism, including regions in the default mode network (DMN), were selected to compare data characteristics with simulation scenarios [35, 36].

## Results

### Simulation data

Table 1 displays the results of the simulation scenarios with *ρ* = 0.3. Based on the identity scaled structure, the power was above 0.90 in sample size *N*= 25, regardless of the heterogeneity amount and the number of regions. In the number of regions above 20 and the sample size of 10 and 25, the type I error rates formed on identities scaled and compound symmetry structures were close to 0.05. Based on the compound symmetry structure, the model had acceptable power values without affecting the degree of heterogeneity in the sample size *N*=25 and *V* > 20.

**Table 1:** Type I error and power of the model to check differentially expressed FC patterns between two groups in term of simulation setting: sample size N, structure of between-subject variability, number of regions *V*, degree of heterogeneity and dependence among the edges *ρ* = 0.3

***p* = 0.3**			Compound Symmetry		Scaled	
			Heterogeneity		Heterogeneity	
	*V*	N	Low	High	Low	High
**Type** ** I ** **Error**	20	10	0.090	0.060	0.090	0.090
		25	0.050	0.050	0.030	0.050
	25	10	0.020	0.000	0.050	0.050
		25	0.050	0.010	0.040	0.040
	30	10	0.060	0.060	0.050	0.050
		25	0.070	0.060	0.070	0.070
**Power**	20	10	0.290	0.280	0.510	0.510
		25	0.550	0.500	0.980	0.960
	25	10	0.470	0.480	0.610	0.590
		25	0.920	0.890	1.000	1.000
	30	10	0.650	0.650	0.760	0.750
		25	0.990	0.960	1.000	1.000

Table 2 shows the results of the simulation scenarios with *ρ* = 0.5. In a sample size N=25, the power based on both structures of between-subject variability was greater than 0.8, regardless of the number of regions and heterogeneity. The model demonstrated acceptable values of power in the identity scaled structure when *N*=10 and *V* = 30. The type I error was close to 0.05 in most scenarios for the region numbers of 20 and 25.

**Table 2:** Type I error and power of the model to check differentially expressed FC patterns between two groups in term of simulation setting: sample size N, structure of between-subject variability, number of regions *V*, degree of heterogeneity and dependence among the edges *ρ* = 0.5

***p* = 0.5**			Compound Symmetry		Scaled	
			Heterogeneity		Heterogeneity	
	*V*	N	Low	High	Low	High
**Type** ** I ** **Error**	20	10	0.050	0.020	0.050	0.070
		25	0.070	0.050	0.040	0.040
	25	10	0.030	0.030	0.050	0.050
		25	0.020	0.030	0.040	0.040
	30	10	0.050	0.060	0.050	0.050
		25	0.100	0.090	0.070	0.070
**Power**	20	10	0.340	0.340	0.660	0.650
		25	0.870	0.960	0.990	0.990
	25	10	0.420	0.430	0.750	0.730
		25	0.980	0.990	1.000	1.000
	30	10	0.640	0.680	0.900	0.880
		25	0.990	1.000	1.000	1.000

Table 3 shows the results of the simulation scenarios with *ρ* = 0.7. Based on the identity scaled structure, the power was above 0.80, regardless of the number of regions, heterogeneity, and sample size. However, according to the compound symmetry structure, the model had lower power in most scenarios. In more than half of the simulation items, the type I error was less than or equal to 0.05.

**Table 3:** Type I error and power of the model to check differentially expressed FC patterns between two groups in term of simulation setting: sample size N, structure of between-subject variability, number of regions *V*, degree of heterogeneity and dependence among the edges *ρ* = 0.7

***p* = 0.7**			Compound Symmetry		Scaled	
			Heterogeneity		Heterogeneity	
	*V*	N	Low	High	Low	High
**Type** ** I ** **Error**	20	10	0.050	0.020	0.070	0.070
		25	0.040	0.010	0.040	0.030
	25	10	0.030	0.060	0.050	0.050
		25	0.080	0.060	0.040	0.040
	30	10	0.030	0.050	0.070	0.080
		25	0.080	0.050	0.070	0.070
**Power**	20	10	0.070	0.050	0.910	0.900
		25	0.150	0.250	1.000	1.000
	25	10	0.100	0.110	0.880	0.870
		25	0.190	0.400	1.000	1.000
	30	10	0.200	0.260	1.000	1.000
		25	0.600	0.770	1.000	1.000

In summary, the power based on identity scaled structure in a sample size of 25 exhibited values greater than 0.90 without influencing the degree of correlation, heterogeneity, and the number of regions. This index had values above 0.80 in the small sample size and *ρ* = 0.7. In most scenarios, the type I error was near to the nominal level. With increasing the number of regions in the sample *N* = 25 and *ρ*= 0.3,0.5, the power based on compound symmetry structure was greater than 0.80. When there was a high correlation and a small sample size, however, the model yielded lower power. In most simulation items with high heterogeneity, the type I error based on compound symmetry structure was near to the nominal level.

### Autism data

The proposed model in the current study was used to compare brain connectivity patterns between healthy individuals and patients with autism. Firstly, the estimation correlation coefficient and clustering of brain regions were calculated using the nonparametric Bayesian method to consider the network topological features. In this method, the brain network regions were divided into four clusters, K = 4, which the largest cluster consisted of 14 regions. Then the covariance matrix with dimensions of 435 × 435 was obtained using the dependency structure of clusters. Following that, the FC patterns between the patient and healthy groups were compared by considering the effect of heterogeneity. Based on compound symmetry and identity scaled structures, the test statistic values were 20.28(*p* = 0.517) and 21.68(*p* = 0.457), respectively. These results indicate no significant difference in entire FC networks between patients and healthy individuals. Additionally, the Pard algorithm proposed by Chen et al. (2015) and the aSPU method proposed by Pan et al. (2014) were used to examine the differences in FC patterns [10, 37]*.* These models also displayed no significant FC networks between the two groups.

**Fig. 2:**
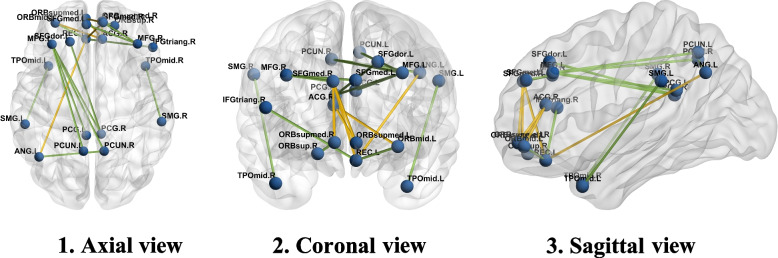
Differentially expressed edges by proposed method: green edges show a connectivity increase between regions of the brain of healthy individuals compared to the patient group, and yellow edges show a connectivity decrease

The FC comparisons of the pair ROIs were performed using test statistics of Eq. (6). Figure 2 depicts the different edges between the healthy and patient groups in three forms: axial, coronal, and sagittal views. In this chart, the increase of functional relationship of the healthy individuals compared to autism patients is manifested with a green edge, and the decrease of functional relationship is manifested with a yellow edge. The results showed out of 435, 18 edges were significant using both structures of variability. However, due to the high number of multiple comparisons, the *p*-value was corrected using the FDR approach. Therefore, after *p*-values adjustment, there was no substantial difference in connectivity between the two groups. Table 4 provides more details of connectivity changes between brain regions.

**Table 4:**
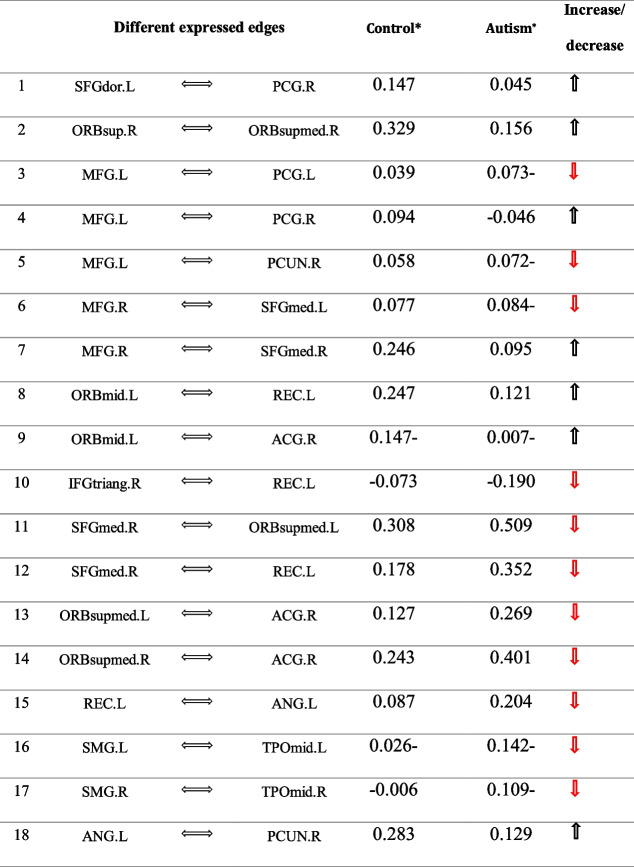
Differentially expressed edges by proposed method; the  symbol shows a connectivity increase between brain regions in healthy individuals compared to the patients group. The  symbol shows a connectivity decrease.

^*^Parameter estimation of the edges between regions in patient and healthy group

Further details on the full names of the regions are available in the appendix.

## Discussion

### Simulation data

To perform statistical inference on FC data, it seems necessary to consider the properties of heterogeneity between subjects and the dependency structure among edges. In previous studies, the spatial closeness between the regions defines the spatial structure [13–16, 38]. Accordingly, given that four regions define a pair of edges, it is difficult to consider the distance between the edges in statistical models. On the other hand, the dependency between the edges is not necessarily limited to the spatial adjacency of the four regions. It can be related to the network topological structure. Recently, Chen et al. (2020) proposed a nonparametric Bayesian model based on network topological features to model the dependency structure of edges. However, this model does not include the heterogeneity effect between subjects which leads to accurate estimation of the parameters. The statistical model proposed in the present study investigates the FC network equality between the patient and healthy groups by combining the nonparametric Bayesian model for estimating the dependency structure between the edges and the algorithm proposed by Fiecas et al. (2017) for considering the heterogeneity of subjects [23, 24].

Simulation data showed that the power improves by increasing the correlation between the edges, based on identity scaled matrix structure. Accordingly, the Bayesian model proposed by Chen et al. (2020) had high accuracy when the correlation was above 0.3 [23]. These results indicate that model performance increases when detecting the network topological structure at higher correlations.

In the compound symmetry structure, the power was affected by the number of regions, sample size, and the edges correlation. Based on compound symmetry and identity scaled structures, the type I error was near to the nominal level when data dimensions and heterogeneity were high. In this regard, Fiecas et al. (2017) presented a variance component model by applying heterogeneity between subjects and temporal dependency, whose performance was similar to the proposed model based on compound symmetry structure in terms of statistical power and type I error. However, in the proposed model, the power based on identity scaled structure yielded a higher value than the variance component model study without limiting the amount of heterogeneity and the number of regions in the sample size of 25 [24]. Accordingly, correct modelling of the dependency structure between the edges can improve the accuracy of estimating the model parameters and the power of the statistical test.

In FC studies, the scale of inference (i.e., at the edge, cluster or network scale) can significantly affect the results of statistical tests. It was recently shown that network-based analysis improves the power to capture average-size effects [39]. Consequently, it is important that the proposed model includes levels of inference from both the network and individual edges.

**Fig. 3:**
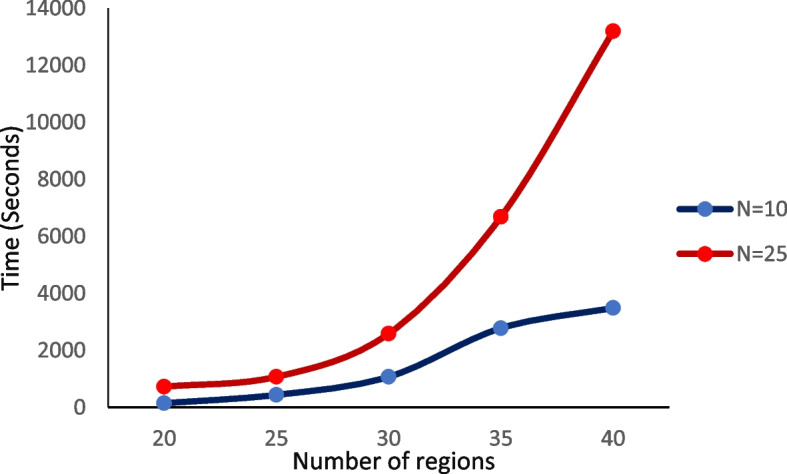
Computation time for the permutation test in various dimensions and the sample sizes of 10 and 25

Another advantage of the proposed model compared to Fiecas et al.’s (2017) model is the improvement of computational power from 190 to 435 edges. However, since the interactions between the brain regions in high dimensions often show network topological features, applying more than 30 regions is one of the model’s limitations due to the high computational cost and time. Figure 3 shows the computational time of the permutation test in different dimensions and the sample size of 10 and 25 on a computer with an i8 CPU and 16G RAM. In order to avoid computing difficulties, structures with a small number of parameters, including the identity scaled and compound symmetry, were used in this study to estimate the between-subject covariance. Therefore, any covariates were not considered in the model. On the other hand, Pard and aSPU models are some of the existing methods for evaluating FC networks, with no limitations in the dimensions of connectivity data [10, 37]. Since there are different patterns of functional connectivity for each subject [29], the heterogeneity effect between subjects is not included in these models.

### Autism data

Estimating the FC patterns can provide a better understanding of the pathologies related to neurodegenerative diseases such as autism, and thus is essential in clinical research. The proposed statistical model in the current study was applied to resting-state fMRI data obtained on 25 patients with autism and 25 healthy individuals. There was no substantial difference in entire FC networks between the patient and healthy groups, which was consistent with the findings of the models proposed by Pan et al. (2014) and Chen et al. (2015) [10, 37]. These results contradict the results of previous studies examining the functional connectivity patterns of autistic patients compared to healthy individuals, which could be due to a large number of regions and the high sample size investigated in these studies [35, 40, 41]. The proposed model is applicable to the analysis of whole-brain connectivity data in practice. However, when there were more than 30 regions, the model’s only shortcoming was the high computation time (Fig. 3). The model can be used with large sample sizes as well. Nevertheless, in order to compare real data characteristics with the simulated scenarios, a sample size of 25 was considered for each patient and healthy group.

Another hypothesis in the proposed statistical model is the FC comparisons of pair regions. In this regard, the permutation test showed the difference in the functional connectivity of several regions between patients and healthy groups regardless of *p*-value adjustment. Most of these changes were related to DMN network regions, including frontal superior medial, cingulum (anterior and posterior), praecuneus, and angular. For example, in the patient group, there was a connectivity decrease of the right posterior cingulate region with the left superior frontal (dorsolateral), left middle frontal regions, and the right anterior cingulate region with the left middle frontal (orbital part). On the other hand, there was an increase in the dependency amount of the right frontal superior medial with the left superior frontal gyrus (medial orbital), and the left angular with the left rectus. Accordingly, several studies have reported functional connectivity changes of DMN regions in autism patients. [42–44]. DMN is one of the most important brain networks whose function changes under the influence of neurological disorders such as autism. These functional changes have a substantial impact on cognitive functions. [45]*.*

## Conclusion

This study presents a new approach for determining differentially expressed FC patterns between two groups, in which heterogeneity between subjects and the structure of dependency among edges are simultaneously considered. Simulation data indicated the high power and near-nominal type I error rates in the proposed model. Additionally, the application of the model on real data related to autism was also evaluated, and there was no significant difference in the functional connectivity network between the patient and healthy groups.

## Supplementary Information

**Additional file 1.**

## Data Availability

The corresponding author can provide the codes of the method upon reasonable request. The data presented in this study are available from the ABIDE database at http://preprocessed-connectomes-project.org/abide/.

## Abbreviations

fMRI: Functional magnetic resonance imaging
FC: Functional connectivity
BOLD: Blood-oxygen-level-dependent
FDR: False discovery rate
ROI: Region of interest
MNI: Montreal Neurological Institute
AAl: Automated anatomical labelling
MCMC: Markov chain Monte Carlo
FIQ: Full intelligence quotient
DMN: Default mode network
