# Supplementary material for: Group-level comparison of brain connectivity networks
Source: BMC Med Res Methodol. 2022 Oct 17;22:273. doi: 10.1186/s12874-022-01712-8 (PMC9575214; doi:10.1186/s12874-022-01712-8)
Supplement: Supplementary file 1 — Additional file 1. [file 12874_2022_1712_MOESM1_ESM.docx]

# Appendix

# Table A1. Target Anatomical Areas according to the AAL Atlas

| Regions | Abbr. | Index |
| --- | --- | --- |
| superior frontal gyrus, dorsolateral, Left | SFGdor.L | 3 |
| Superior frontal gyrus, orbital part, Right | ORBsup.R | 6 |
| Middle frontal gyrus, Left | MFG.L | 7 |
| Middle frontal gyrus, Right | MFG.R | 8 |
| Middle frontal gyrus, orbital part, Left | ORBmid.L | 9 |
| Inferior frontal gyrus, triangular part, Left | IFGtriang.L | 13 |
| Inferior frontal gyrus, triangular part, Right | IFGtriang.R | 14 |
| Rolandic operculum, Right | ROL.R | 18 |
| Olfactory cortex, Left | OLF.L | 21 |
| Superior frontal gyrus, medial, Left | SFGmed.L | 23 |
| Superior frontal gyrus, medial, Right | SFGmed.R | 24 |
| Superior frontal gyrus, medial orbital, Left | ORBsupmed.L | 25 |
| Superior frontal gyrus, medial orbital, Right | ORBsupmed.R | 26 |
| Gyrus rectus, Left | REC.L | 27 |
| Insula, Left | INS.L | 29 |
| Insula, Right | INS.R | 30 |
| Anterior cingulate and paracingulate gyri, Left | ACG.L | 31 |
| Anterior cingulate and paracingulate gyri, Right | ACG.R | 32 |
| Posterior cingulate gyrus, Left | PCG.L | 35 |
| Posterior cingulate gyrus, Right | PCG.R | 36 |
| Middle occipital gyrus, Left | MOG.L | 51 |
| Supramarginal gyrus, Left | SMG.L | 63 |
| Supramarginal gyrus, Right | SMG.R | 64 |
| Angular gyrus, Left | ANG.L | 65 |
| Angular gyrus, Right | ANG.R | 66 |
| Precuneus, Left | PCUN.L | 67 |
| Precuneus, Right | PCUN.R | 68 |
| Superior temporal gyrus, Right | STG.R | 82 |
| Temporal pole: middle temporal gyrus, Left | TPOmid.L | 87 |
| Temporal pole: middle temporal gyrus, Right | TPOmid.R | 88 |

Abbr: abbreviations. L and R correspond to left (L) and right (R)
